# Health systems, policies and infant mortality in developing countries

**DOI:** 10.1186/1471-2458-12-S2-A23

**Published:** 2012-11-27

**Authors:** David Baguma, Jamal Hisham Hashim, Syed Mohamed Aljunid

**Affiliations:** 1 United Nations University International Institute for Global Health, Universiti Kebangsaan Malaysia Medical Centre, Jalan Yaacob Latiff, 56000 Kuala Lumpur, Malaysia

## Background

The global movement to improve the quality of life advances the role of health systems and their policies to reduce challenges faced such as health risks caused by climate change and environmental hazards. In this paper, we focus on infant mortality and examine the status of the infant mortality between birth and one year age in developing countries.

## Methods

The secondary data on infant mortality was obtained on countries in Eastern Africa, Western Europe and Southern Asian countries between 1950 and 2010 expressed as deaths per 1,000 births. Countries in Western Europe were used for comparison purposes. The data also included inpatients (admissions and deaths in 2007) of water-related diseases such as malaria and diarrhoea, in Eastern Africa (i.e., Uganda). Nonlinear regression modelling was utilized in the empirical analysis.

## Results

The study shows that, children deaths between birth and age one had declined in Eastern Africa by 7%, Western Europe by 23% and reduced by 15% in Southern Asian countries. The study also shows that the Eastern African countries require more than 50 years to improve infant mortality to levels close to Western Europe countries (i.e., 3.52 > 3.6), ceteris-paribus.

## Conclusion

Inadequacies in the health systems, climate change linked health risks, low national incomes and water-related diseases are partly some of the causes of high infant mortality in which policy measures could emphasize for global health interventions.

